# miR-211 Plays a Critical Role in *Cnidium officinale* Makino Extract-Induced, ROS/ER Stress-Mediated Apoptosis in U937 and U266 Cells

**DOI:** 10.3390/ijms19030865

**Published:** 2018-03-15

**Authors:** Jin Ah Cha, Hyo-Sook Song, Beomku Kang, Moon Nyeo Park, Kyoung Sun Park, Sung-Hoon Kim, Bum-Sang Shim, Bonglee Kim

**Affiliations:** 1 Department of Pathology, College of Korean Medicine, Graduate School, Kyung Hee University, 1 Hoegi-dong, Dongdaemun-gu, Seoul 130-701, Korea; nicesh89@khu.ac.kr (J.A.C.); beomkukang@khu.ac.kr (B.K.); mnpark@khu.ac.kr (M.N.P.); sungkim7@khu.ac.kr (S.-H.K.); 2 Department of Science in Korean Medicine, College of Korean Medicine, Graduate School, Kyung Hee University, 1 Hoegi-dong, Dongdaemun-gu, Seoul 130-701, Korea; shs331@khu.ac.kr; 3 Department of Korean Medicine Obstetrics & Gynecology, College of Korean Medicine, Kyung Hee University, 1 Hoegi-dong, Dongdaemun-gu, Seoul 130-701, Korea; lovepks@khnmc.or.kr

**Keywords:** *Cnidium officinale* Makino, endoplasmic reticulum stress, C/EBP homologous protein, reactive oxygen species, miR-211

## Abstract

Though *Cnidium officinale* Makino (COM) was known to have anti-angiogenic, anti-oxidant, neuroprotective, and anti-cancer effects, the underlying anticancer mechanism of COM using endoplasmic reticulum (ER) stress and miRNA remained unclear until now. Thus, in the current study, the inhibitory mechanism of COM in lymphoma and multiple myeloma (MM) cells was elucidated. COM exerted cytotoxicity in U937 and U266 but not Raw264.7 cells. COM treatment increased the expression of ER stress-related proteins such as p-protein kinase RNA-like endoplasmic reticulum kinase (p-PERK), p-eukaryotic initiation factor (p-eIF2α), and activating transcription factor 4 (ATF4) and C/EBP homologous protein (CHOP). COM also cleaved poly (ADP-ribose) polymerase (PARP) in a dose-dependent manner in both cells. Also, reactive oxygen species (ROS) generation was elevated by COM treatment. Conversely, the apoptotic effect of COM treatment was blocked by *N*-acetyl-l-cysteine (NAC) pretreatment. Also, the pro-survival miRNA, miR-211 was decreased by COM treatment in U937 and U266 cells. miR-211 mimic attenuated COM-induced apoptosis. Taken together, these results support the scientific evidence that COM induces apoptosis via ROS generation/CHOP activation and miR-211 suppression in U937 and U266 cells.

## 1. Introduction

Multiple myeloma is a hematological cancer that develops mainly in monoclonal plasma cells. The mortality rate of multiple myeloma is less than five years; in the case of acute, severe MM, it is just over two years [1]. There has been continuous research into novel therapies, such as immunomodulatory drugs, proteasome inhibitors, and monoclonal antibodies, but the limitations of such approaches are evident at the moment [2]. Thus, new mechanisms that have potent anti-MM activities are needed; in this study, we have focused on endoplasmic reticulum stress (ER stress) as a potential mechanism for combatting multiple myeloma.

The endoplasmic reticulum (ER) is an intracellular organelle related to various cellular protein activities. Contemporary research takes interest in a phenomenon in the ER called endoplasmic reticulum stress (ER stress). Recently, there has been research into ER stress as an agonist or antagonist in pathological conditions—mostly related to cellular dysfunction and cell death [3]. This is mainly why researchers are currently focusing on controlling ER stress-related pathways as therapeutic targets of pharmacological research. ER stress is initiated by external stimuli such as hypoxia, oxidative stress, and calcium homeostasis disruption [4]. ER stress triggers a process called the unfolded protein response (UPR). UPR is mainly classified into three procedures if disequilibrium between protein folding and the entry of unfolded protein is detected by BiPs or UGGTs [5]. The following steps occur during the UPR process: (1) protein levels entering the ER are reduced; (2) protein folding mechanisms are stimulated; (3) if protein homeostasis cannot be achieved in either of the first two steps, apoptosis is induced in unfolded proteins by an endoplasmic reticulum-associated protein degradation (ERAD) process [6]. These three steps of UPR are usually carried out by three major transcriptional factors: (1) IRE1α; (2) ATF6; and (3) PERK. When these factors fail to alleviate ER stress, intrinsic/extrinsic pathways of apoptosis are often initiated, leading to programmed cell death [7]. In ER stress, various factors mediate such mechanisms; for example, proteases (especially caspases), kinases, transcription factors (CHOP), and the Bcl-2 family proteins [8]. In this study, the main focus was ROS generation, which takes part in the induction of apoptosis in human multiple myeloma cell lines. There have been studies targeting the ER stress-mediated pathways in multiple myeloma treatment. Toyocamycin—derived from *Streptomyces* sp., a genus of actinobacetria—was observed to be an inhibitor of ER stress-induced XBP1 mRNA splicing and the selective inhibition of the IRE1α-XBP1 pathway [9]. Also, a proteasome inhibitor (PI) named bortezomib has been shown to initiate UPR in multiple myeloma cell lines by expressing certain ER chaperones and inducing the expression of transcription factors associated with ER stress-induced apoptosis [10]. As seen above, there are many factors inducing ER stress and UPR that finally induce apoptosis and cell death. Often, the three major transcriptional factors and the factors mentioned above are targets of research. In many cases, oxidative stress conditions are observed with ER stress, and current research has revealed that PERK (RNA-dependent protein kinase (PKR)-like ER kinase), a major factor in UPR, contributes to apoptosis by controlling the levels of pro-apoptotic C/EBP homologous protein (CHOP) and facilitating the propagation of ROS signals between the ER and the mitochondria [11].

MicroRNAs repress target gene transcription at the level of posttranscriptional gene silencing [12]. The role of microRNAs in ER stress responses has recently been studied. Among the ER stress-related microRNAs, miR-211 is reported to work as a pro-survival miRNA, preventing pro-apoptotic transcription factor CHOP accumulation [13].

To find the anti-cancer effects of medical herbs against myeloid-originated hematological malignancies, a screening test was conducted with *Cnidium officinale* Makino (COM), *Salvia miltiorrhiza* (SM), *Achyranthes bidentata* Blume (ABB), *Eupolyphaga sinesis* Walker (ESW), and *Hirudo nipponica* Whitman (HNW) (Supplementary Figures S1 and S2). ABB, ESW and HNW treatment did not show significant cytotoxicity up to 200 µg/mL in U937 and U266 cells. However, SM and COM showed an apoptotic effect against U937 and U266 cancer cells. The results of SM have been submitted as a separate manuscript [14].

*Cnidium officinale* Makino (COM) has been used as a traditional medicine for thousands of years in Korean, China, and Japan for treating blood-related diseases such as blood stasis, contusion, and infertility. Recently, COM has been reported to have an anti-cancerous effect in liver cancer [15], colorectal cancer [16], and oral cancer [17]. However, the effect of COM against multiple myeloma has not been elucidated yet. Thus, in this study, the aim is to reveal the mechanism of COM. In this study, we first illuminate COM’s effect on inducing apoptosis via ROS/ER stress and miR-211 regulated apoptotic mechanisms.

## 2. Results

### 2.1. COM Exerted Cytotoxicity in U937 and U266 Cells

**Figure 1:**
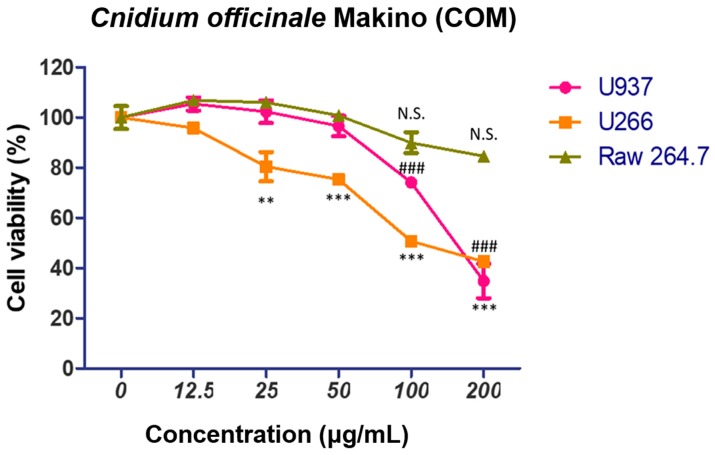
Cytotoxic effect of *Cnidium officinale* Makino in U937 and U266 cells. U266 and U937 cells and murine macrophage cell lines Raw264.7 were seeded onto 96-well microplates at a density of 2 × 10^4^ cells/well and treated with various concentrations of COM (0, 12.5, 25, 50, 100 or 200 μg/mL) for 24 h. Cell viability was determined by EZ-cytox Enhanced cell viability assay kit. Values represent the means of three experiments ± SD; ** *p* < 0.01, *** *p* < 0.001 versus untreated control of U266; ^###^ *p* < 0.001 versus untreated control of U937. N.S. means not significant.

To demonstrate the cytotoxic effect of COM against multiple myeloma cells (U266), myeloid lymphoma cells (U937), and mouse normal macrophage cells (Raw264.7), a cytotoxicity assay was conducted. Cells were treated with various concentrations of COM (0, 12.5, 25, 50, 100, or 200 μg/mL) for 24 h and the assay was performed. As shown in Figure 1, COM notably decreased the cell viability of U937 and U266 cells in a dose-dependent manner, but not Raw264.7 cells.

### 2.2. COM Treatment Increased ER Stress-Related Proteins in U937 and U266 Cells

**Figure 2:**
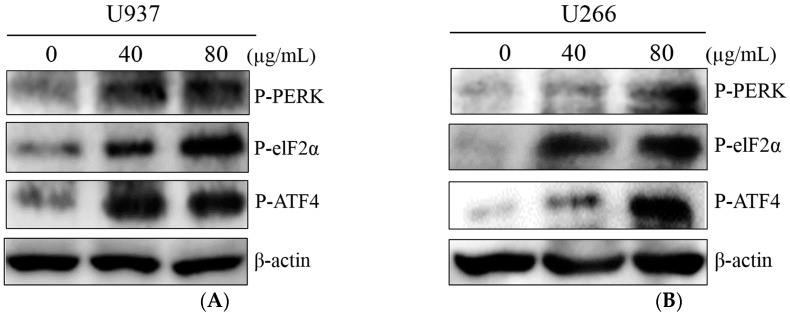
The effect of COM on ER stress-related proteins in U937 and U266 cells. (**A**) U937 cells were treated with COM (40 or 80 μg/mL) for 24 h, and Western blot analysis was performed for P-PERK, P-eIF2α, P-ATF4, and β-actin; (**B**) U266 cells treated under the same conditions.

Recently, ER stress is reported to have essential roles in cancer cell death [18]. To elucidate the mechanism of the COM-induced cytotoxicity, the expression of ER stress-related proteins (p-PERK, p-eIF-2α, ATF4) were measured by Western blot analysis. U937 and U266 cells were exposed to COM for 24 h. COM treatment increased the expression of p-PERK, p-eIF-2α, and p-ATF4 at a concentration of 40 and 80 μg/mL (Figure 2A,B).

### 2.3. COM Treatment Induced Apoptosis in U937 and U266 Cells

**Figure 3:**
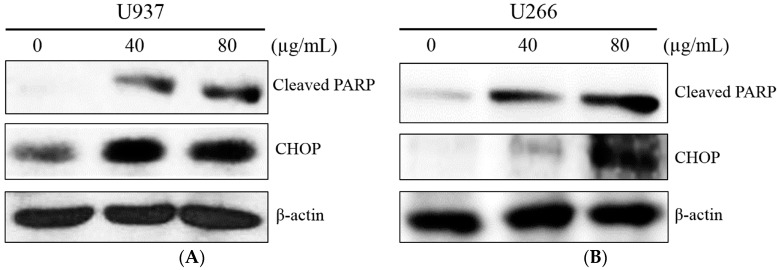
The effect of COM on ER stress-mediated apoptosis-related proteins in U937 and U266 cells. (**A**) U937 cells were treated with COM (40 or 80 μg/mL) for 24 h, and Western blot analysis was performed for cleaved PARP, CHOP, and β-actin; (**B**) U266 cells treated under the same conditions, Western blot analysis.

An ER stress transcription factor, CHOP, induces apoptosis in response to cellular stress [19]. To confirm the ER stress induction by COM treatment triggered apoptosis, Western blot analysis for CHOP and cleaved PARP was performed. COM (40 or 80 μg/mL) was administered to U937 and U266 cells for 24 h. As shown in Figure 3A,B, COM treatment increased the expression of CHOP and cleaved PARP in a dose-dependent manner.

### 2.4. COM Treatment Induced Apoptosis via ROS Generation in U937 and U266 Cells

**Figure 4:**
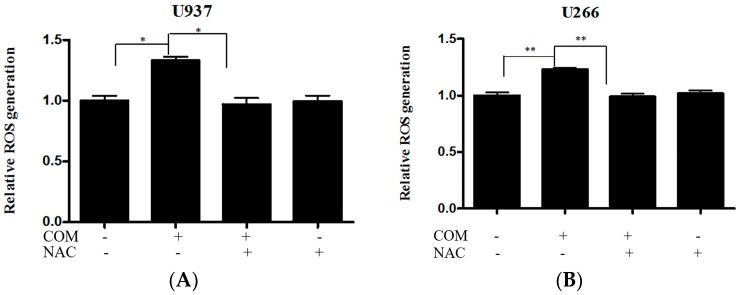
The effect of COM on ROS production in U937 and U266 cells. (**A**) U937 cells were treated with COM (80 μg/mL) for 24 h with or without pre-treatment with NAC (5 mM) for 1 h. ROS production was analyzed using oxidation-sensitive fluorescent dye (DCFDA) by cellular reactive oxygen species detection assay kit; (**B**) U266 cells treated under the same conditions. Values represent the means of three experiments ± SD; * *p* < 0.05, ** *p* < 0.01, versus untreated control.

**Figure 5:**
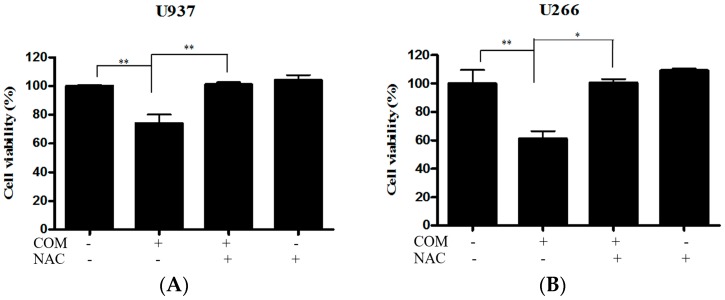
The effect of ROS scavenger on COM-induced apoptosis in U937 and U266 cells. (**A**) U937 cells were treated with COM (80 μg/mL) for 24 h with or without pre-treatment of NAC (5 mM) for 1 h. Cell viability was determined by EZ-cytox Enhanced cell viability assay kit; (**B**) U266 cells treated under the same conditions. Values represent the means of three experiments ± SD; * *p* < 0.05, ** *p* < 0.01, versus COM treated control.

**Figure 6:**
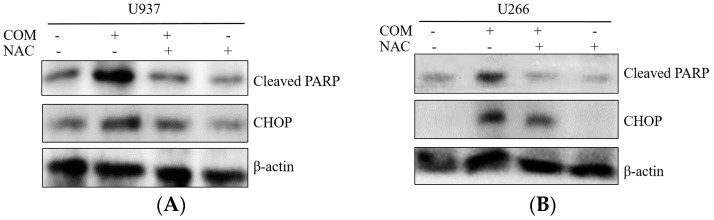
The effect of ROS scavenger on COM-induced PARP cleavage and CHOP activation in U937 and U266 cells. (**A**) U937 cells were treated with COM (80 μg/mL) for 24 h with or without pre-treatment of NAC (5 mM) for 1 h. Cell lysates were prepared and subjected to Western blot analysis for cleaved PARP and CHOP; (**B**) U266 cells treated under the same conditions.

The enhancement of PERK/ATF4 signaling is mediated by ROS generation in apoptosis of cancer cells [20]. To explore the role of ROS generation in COM-induced apoptosis, DCFDA—cellular reactive oxygen species detection assay kit, cell viability assay, and Western blot analysis were conducted with COM-exposed U937 and U266 cells. COM treatment significantly increased ROS generation compared to the control group (by about 33% and 23% in U937 and U266 cells, respectively). To investigate the role of ROS in COM-induced apoptosis, the ROS scavenger NAC was administered to the cells as a pretreatment. The increased ROS was decreased by NAC pretreatment, as expected (Figure 4A,B). The cytotoxicity of COM was significantly attenuated by NAC pretreatment compared to the COM-only-treated group (Figure 5A,B). Also, the elevated expression of cleaved PARP and CHOP by COM treatment was reversed by NAC pretreatment (Figure 6A,B).

### 2.5. COM Downregulated the Expression of miR-211 in U937 and U266 Cells

**Figure 7:**
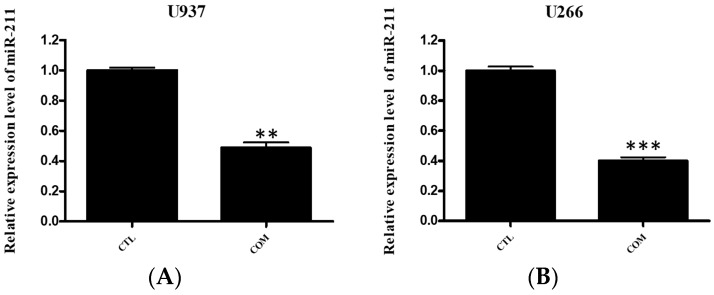
The effect of COM suppressed the expression of miR-211 in U937 and U266 cells. In U937 (**A**) and U266 (**B**) cells, miR-211 levels was determined by a qRT-PCR array after 24 h COM (80 μg/mL) treatment. Values represent the means of three experiments ± SD; ** *p* < 0.01, *** *p* < 0.001, versus untreated control.

Some miRNAs have role in ER stress mediated apoptosis. During the ER stress-induced apoptosis, miR-211 suppresses ER stress pro-apoptotic transcription factor, CHOP. The proximal CHOP promoter is directly targeted by miR-211. miR-211 downregulation is important in CHOP accumulation and induces apoptosis [13]. To determine whether miR-211 is affected by COM treatment, qRT-PCR was performed. Cells were treated with COM (80 μg/mL) for 24 h. As shown in Figure 7A,B, the expression of miR-211 was suppressed by COM treatment in both U937 and U266 cells.

### 2.6. miR-211 Plays a Critical Role in COM-Induced Apoptosis in U937 and U266 Cells

**Figure 8:**
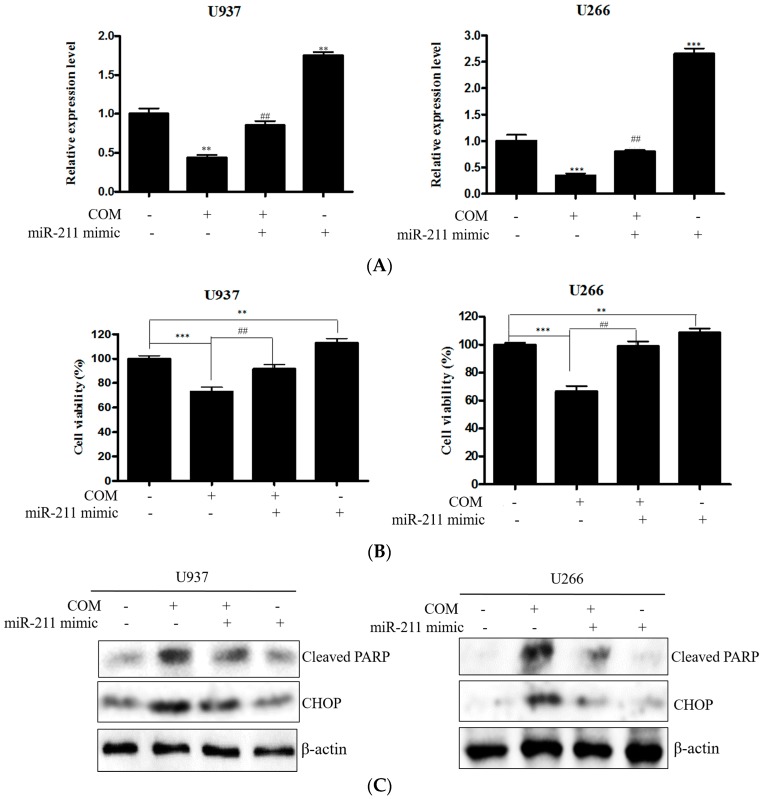
The effect of miR-211 in COM-induced apoptosis in U937 and U266 cells. Cells were transfected with miR-211 mimic for 48 h. (**A**) The expression level of miR-211 was determined by a qRT-PCR array after 24 h COM (80 μg/mL) treatment with or without miR-211 mimic transfection; (**B**) cell viability was determined by EZ-cytox Enhanced cell viability assay kit (80 μg/mL of COM exposure for 24 h); (**C**) Cells were treated with COM (80 μg/mL) for 24 h. Cell lysates were prepared and subjected to Western blot analysis for cleaved PARP and CHOP. Values represent the means of three experiments ± SD; ** *p* < 0.01, *** *p* < 0.001 versus untreated control; ^##^ *p* < 0.01 versus COM treated group.

To confirm the role of miR-211, qRT-PCR and EZ-cytox were conducted with miR-211 mimic transfected cells. U937 and U266 cells were transfected with miR-211 mimic for 48 h and exposed to 80 μg/mL of COM for 24 h. The expression of miR-211 was significantly increased in the miR-211 mimic-transfected group compared to the untreated control. Decreased expression of miR-211 was caused by miR-211 mimic transfection (Figure 8A). As shown in Figure 8B, the viability of miR-211 mimic transfected cells was elevated in both U937 and U266 cells. Also, the suppressed viability of COM-treated cells was ameliorated in miR-211 mimic transfected cells. To identify the mechanism, Western blot analysis was experimented. The increased expression of cleaved PARP and CHOP was attenuated by the miR-211 mimic (Figure 8C).

## 3. Discussion

Multiple myeloma is the second most frequent hematological malignancy. During the last 20 years, advances in treatment have had a marked impact on the survival of patients with multiple myeloma. Melphalan was the first treatment for myeloma treatment in 1969 [21]. High-dose chemotherapy with autologous stem cell transplantation was conducted until new drugs, including thalidomide [22] and bortezomib [23], were developed and showed significant survival advantages. MM-induced death rates have decreased recently due to novel drugs, with a five-year survival rate of 48.5% [24]. However, MM is still a grave hazard in hematomas, and currently, fervent research is being undertaken in areas that target apoptosis in multiple myeloma cell lines.

Traditional medicines have been used for thousands of years to treat all kind of diseases, including cancer, around the world. In Korea, traditional medicine is still used under the supervision of Ministry of Health and Welfare of Korea, by licensed traditional doctors. There are categorized lists of traditional medicines, named Hwal-Hyeol-Geo-Yeo (HHGY) herbs, including *Cnidium officinale* Makino (COM), *Salvia miltiorrhiza* (SM), *Achyranthes bidentata* Blume (ABB), *Eupolyphaga sinesis* Walker (ESW), *Hirudo nipponica* Whitman (HNW), etc. These herbs were traditionally used to improve blood circulation and decrease the viscosity of blood. Hematological cancer induces a hypercoagulability state in 82.7% of patients with one or more markers of thrombosis, which increases morbidity and mortality [25]. In that respect, the HHGY herbs could be effective anti-MM drug candidates. Thus, the anti-MM effects of HHGY herbs were investigated in this study. COM showed anti-MM properties via ROS generation-dependent, ER stress-induced apoptosis, similar to SM. However, there are differences in epigenetic mechanisms between COM and SM. COM suppressed one of the onco-miRNAs, miR-211, which in turn targeted and repressed CHOP, while SM induced apoptosis by miR-216b upregulation, which attenuated C-Jun expression [14]. This difference could be a great opportunity in that a combination of COM and SM might be more powerful and have a synergistic effect by regulation of both miRNAs. Also, those two herbal medicines have traditionally been used for blood-related diseases. This study is ongoing and further mechanisms that rely on a combination of COM and SM are being investigated.

*Cnidium officinale* Makino (COM) has anti-retinal neovascularizational effect [26], anti-inflammatory effect [27], and anti-angiogenic effect [28]. Also, COM has anti-cancerous effect against liver cancer (by regulation of caspase-3, p53, Bcl-2) [15], colorectal cancer (by modification of p53, p21, Bax and caspase-3) [16], and oral cancer (by modulation of PARP, Mcl-1) [17]. Although there have been various studies about specific mechanisms of apoptosis, here we illuminate for the first time the apoptosis mechanism of COM via ER stress and miRNA regulation against MM.

To check whether COM played a role in exerting cytotoxicity in multiple myeloma cell lines, we conducted a cell viability assay in U937 and U266 cells. The results displayed a notable decrease in the overall cell viability percentage. This demonstrated that COM is indeed a main exhibitor of cytotoxicity. However, in normal macrophage cells line, Raw264.7 cells’ decrease in cell viability was minimal, so we concluded that COM exerted remarkable cytotoxic effects only in U937 and U266 cancer cells.

In COM-induced programmed cell death, we sought to determine whether ER stress factors were associated. In order to clarify this, we used Western blot analysis to test whether the levels of ER stress-related proteins such as p-PERK, p-eIF-2α, and ATF4 were altered in the process of inducing cell death. Inferring from Figure 2A,B, the increased expression of p-PERK, p-eIF-2α, and ATF4 was notable. This suggests that ER stress-related proteins were activated, thus confirming that ER stress-related proteins play a role in inducing cell death. From Figure 3A,B, we deducted that the increase of ER stress transcription factor CHOP and cleaved PARP influenced the upregulation of ER stress. Taking into account the results of the cell viability assay and Western blot analysis, we concluded that COM may induce apoptosis via the ER stress mechanism.

There has been ongoing research about the cross-talk between ROS generation and ER stress. We have concluded from previous studies that some forms of ROS trigger ER stress, while others do not [29]. In order to clarify whether ROS generation is related to COM-induced apoptosis via ER stress, U937 and U266 have undergone ROS measurement assay, cell viability assay, and Western blot analysis. Significant increase in ROS generation was observed in COM-treated cell lines. Thus we hypothesized that ROS generation might play a role in inducing apoptosis via ER stress.

However, to confirm that COM was fully responsible for generating ROS in U937 and U266 cell lines, we pretreated the cells with NAC and conducted an ROS scavenger test. This was necessary because there might have been other factors that generated ROS, and we needed to confirm that COM was the main generator of ROS. So we compared COM-treated cells with COM+NAC treated cells. The results in the COM+NAC treated cells, as expected, showed a decrease in ROS generation. This was an indicator that COM was indeed the mediator of ROS generation. Thus, we concluded that COM was the major factor that generated ROS, then inducing ER stress and thus inducing apoptosis.

Finally, the relationship of miRNA and ER-stress-induced apoptosis in COM-induced apoptosis was studied. Recently it was reported that miR-211 suppressed the ER stress pro-apoptotic factor, CHOP [13]. By performing qRT-PCR in COM-treated cells, we confirmed that downregulation of miR-211 occurred in both U937 and U266 cells. Also, miR-211 mimic transfection suppressed the apoptotic effect of COM, indicating that miR-211 repression is a pivotal mechanism of COM-induced apoptosis.

In this study, we have concentrated on miR-211, which plays a role in inducing apoptosis via ER stress. However, there has been previous research into other miRNAs that might influence ER stress. For example, miR-455 influences levels of IRE1 to induce ER stress [30]. Also, the overexpression of miR-122 was found to repress the activation of the UPR pathway, which is directly related to ER stress-inducing mechanisms [31]. Furthermore, miR-221/222 was reported to resist cell death by sensitizing the cell to ER stress-induced apoptosis [32]. Further research in the area of miRNAs and ER stress should be facilitated, to fully elucidate the nature of miRNA regulation and cell activity.

Overall, our study has elucidated new mechanisms of COM-induced apoptosis in U937 and U266 cell lines: apoptosis via ER stress, which is influenced by ER stress-related proteins and ROS generation, and by regulating miR-211, which is associated with CHOP, a pro-apoptotic transcription factor. Evaluations of ER stress-related factors/miRNAs that trigger apoptosis should be further ascertained.

## 4. Materials and Methods

### 4.1. Chemicals and Reagents

*Cnidium officinale* Makino (Beijing, China) (1 kg) was identified by Prof. Minho Lee of the Department of Oriental Medicine Biotechnology, Kyung Hee University (Seoul, South Korea). A voucher specimen (No. KH-00107) was maintained at the herbarium of the Department of Pathology, College of Korean Medicine, Kyung Hee University. Then, COM was extracted with 99.9% EtOH and extracted solutions were filtered and evaporated to produce EtOH. The lyophilized COM extract was then made into a powder form. It was dissolved in dimethyl sulfoxide (DMSO) as a stock solution (400 mM) and stored at −20 °C.

### 4.2. Cell Culture

Human myeloid lymphoma U937 cells and murine macrophage Raw264.7 cells were obtained from the Korean Cell Line Bank (Seoul, Korea). Human multiple myeloma U266 cells were from the American Type Culture Collection (Manassas, VA, USA). They were routinely maintained in RPMI 1640 (Welgene, Daegu, Korea), supplemented with 10% FBS and penicillin/streptomycin, and grown at 37 °C in a humidified atmosphere of 5% CO_2_.

### 4.3. Cytotoxicity Assay

The cytotoxicity of *Cnidium officinale* Makino was determined using the EZ-cytox cell viability assay kit (DoGen, Seoul, Korea). U937 and U266 cells were seeded onto 96-well microplates at a density of 2 × 10^5^ cells per well and treated with various concentrations of *Cnidium officinale* Makino (0, 12.5, 25, 50, 100, or 200 μg/mL) for 24 h with or without pre-treatment of NAC (5 mM) for 1 h. A volume of 10 μL of EZ-cytox kit solution was added to each well and incubated for another 2 h at 37 °C. Optical density (OD) was measured using a microplate reader (Bio-Rad, Hercules, CA, USA) at 450 nm. Cell viability was expressed as a percentage of the absorbance present in *Cnidium officinale* Makino treated group compared with control cells.

### 4.4. Western Blot Analysis

Cells (1 × 10^6^ cells/mL) were treated with indicated concentrations of *Cnidium officinale* Makino (40 or 80 μg/mL) for 24 h. The cells were subsequently lysed with RIPA buffer (50 mM Tris-HCl, pH 7.4, 150 mM NaCl, 1% NP-40, 0.25% sodium deoxycholic acid, 1 M EDTA, 1 mM Na_3_VO_4_, 1 mM NaF) containing a protease inhibitors cocktail (Amresco, Solon, OH, USA). The protein supernatants were collected and quantified for protein concentration using an RC DC protein assay kit II (Bio-Rad, Hercules, CA, USA). Proteins were separated by SDS-PAGE 8–12% gels and transferred to nitrocellulose membranes. The membranes were blocked with 5% (*v*/*v*) non-fat dried milk in Tris-buffered saline with 0.05% Tween 20 and incubated with the required antibodies. Primary antibodies were used at a 1:500~1000 dilution (5% bovine serum albumin) and secondary antibodies at a 1:1000 dilution (5% skim milk). Then they were transferred to a Hybond ECL (Amersham Pharmacia, Piscataway, NJ, USA) transfer membrane for detection with antibodies for cleaved-PARP: CHOP (Cell Signaling, Danvers, MA, USA), ATF4 (Thermo Fisher, Waltham, MA, USA), P-PERK (Abcam, Cambridge, MA, USA), P-eIF2α (Bioss, Woburn, MA, USA), and β-actin (Santa Cruz, Dallas, TX, USA).

### 4.5. Measurement of ROS Generation

The ROS generation of COM was measured using the DCFDA—Cellular Reactive Oxygen Species Detection Assay Kit (Abcam, Cambridge, MA, USA). U937 and U266 cells were seeded onto 96-well microplates at a final concentration of 1 × 10^5^ cells per well and treated with *Cnidium officinale* Makino (80 μg/mL) for 24 h with or without pre-treatment of NAC (5 mM) for 1 h. After staining the cells in 20 μM DCFDA solution, we incubated them at 37 °C for 30 min in the dark. They were measured on a fluorescence plate reader (Bio-Rad 680, USA) at Ex/Em = 485/535 nm in end point mode in the presence of compounds, media, or buffer. Identical untreated control and NAC-only treated groups were used for screening tests of *Salvia miltiorrhiza* and *Cnidium officinale* Makino.

### 4.6. qRT-PCR

Total RNA was extracted from individual COM-treated samples using the TRIzol extraction method, as described by the manufacturer (Invitrogen, Carlsbad, CA, USA). RNA was reverse-transcribed using a Mir-X miRNA First-Strand Synthesis Kit (Takara Bio, Nojihigashi, Japan) according to the manufacturer’s protocol. Quantitative PCR was conducted using a standard protocol from the SYBR Premix Ex Taq II (Takara Bio, Nojihigashi, Japan). Primer sequences (human) were as follows: mature hsa-miR-211 Forward 5′-TTCCCTTTGTCATCCTTCGC-3′ and U6 primers using (Cat. #638313, Takara Bio, Nojihigashi, Japan).

### 4.7. miRNA Mimic Transfection

The micro RNA mimic transfection was performed using a TransITX2^®^ Dynamic Delivery System kit (Mirus, MD, USA) as per the manufacturer’s protocol. U937 and U266 cells were seeded onto six-well plates at a density of 2.5~5.0 × 10^5^ cells per well and the cell cultures were incubated overnight. Opti-MEM I Reduced-Serum medium was added to 25 nM RNA and 7.5 μL *Trans*IT-X2 and they were gently mixed and incubated at room temperature for 15–30 min. Then, U937 and U266 cells were exposed to has-miR-211-5P mimic COM (80 μg/mL) for 24 h. Primer sequences (human) were as follows: has-miR-211-5P: 5′-UUCCCUUUGUCAUCCUUCGCCU-3′ (Sense), 5′-AGGCGAAGGAUGACAAAGGGAA-3′ (Antisense).

### 4.8. Statistical Analysis

Data were expressed as means ± standard deviation (SD). Statistical significance was determined by Student’s *t*-test and one-way ANOVA test using Sigmaplot version 12 software (Systat Software Inc., San Jose, CA, USA).

## Supplementary Materials

Supplementary materials can be found at http://www.mdpi.com/1422-0067/19/3/865/s1.

## References

[1] Agarwal A., Ghobrial I.M. (2012). Monoclonal gammopathy of undetermined significance and smoldering multiple myeloma: A review of the current understanding of epidemiology, biology, risk stratification, and management of myeloma precursor disease. Clin. Cancer Res..

[2] Barlogie B., Shaughnessy J., Tricot G., Jacobson J., Zangari M., Anaissie E., Walker R., Crowley J. (2004). Treatment of multiple myeloma. Blood.

[3] Kim I., Xu W., Reed J.C. (2008). Cell death and endoplasmic reticulum stress: Disease relevance and therapeutic opportunities. Nat. Rev. Drug Discov..

[4] Hetz C. (2012). The unfolded protein response: Controlling cell fate decisions under ER stress and beyond. Nat. Rev. Mol. Cell Biol..

[5] Schröder M., Kaufman R.J. (2005). ER stress and the unfolded protein response. Mutat. Res. Fundam. Mol. Mech. Mutagen..

[6] Ron D., Walter P. (2007). Signal integration in the endoplasmic reticulum unfolded protein response. Nat. Rev. Mol. Cell Biol..

[7] Sano R., Reed J.C. (2013). ER stress-induced cell death mechanisms. BBA Mol. Cell Res..

[8] Xu C., Bailly-Maitre B., Reed J.C. (2005). Endoplasmic reticulum stress: Cell life and death decisions. J. Clin. Investig..

[9] Ri M., Tashiro E., Oikawa D., Shinjo S., Tokuda M., Yokouchi Y., Narita T., Masaki A., Ito A., Ding J. (2012). Identification of Toyocamycin, an agent cytotoxic for multiple myeloma cells, as a potent inhibitor of ER stress-induced XBP1 mRNA splicing. Blood Cancer J..

[10] Obeng E.A., Carlson L.M., Gutman D.M., Harrington W.J., Lee K.P., Boise L.H. (2006). Proteasome inhibitors induce a terminal unfolded protein response in multiple myeloma cells. Blood.

[11] Verfaillie T., Rubio N., Garg A.D., Bultynck G., Rizzuto R., Decuypere J.P., Piette J., Linehan C., Gupta S., Samali A. (2012). PERK is required at the ER-mitochondrial contact sites to convey apoptosis after ROS-based ER stress. Cell Death Differ..

[12] Kim B., Srivastava S.K., Kim S.H. (2015). Caspase-9 as a therapeutic target for treating cancer. Expert Opin. Ther. Targets.

[13] Chitnis N.S., Pytel D., Bobrovnikova-Marjon E., Pant D., Zheng H., Maas N.L., Frederick B., Kushner J.A., Chodosh L.A., Koumenis C. (2012). miR-211 is a prosurvival microRNA that regulates chop expression in a PERK-dependent manner. Mol. Cell.

[14] Kim C., Song H.-S., Park H., Kim B. (2018). Activation of ER stress dependent-miR-216b has critical role in *Salvia miltiorrhiza* Ethanol Extract Induced Apoptosis in U266 and U937 cells. Int. J. Mol. Sci..

[15] Hong H., An J.C., de la Cruz J.F., Hwang S.G. (2017). *Cnidium officinale* Makino extract induces apoptosis through activation of caspase-3 and p53 in human liver cancer HepG2 cells. Exp. Ther. Med..

[16] De la Cruz J., Kim D.H., Hwang S.G. (2014). Anti cancer effects of *Cnidium officinale* Makino extract mediated through apoptosis and cell cycle arrest in the HT-29 human colorectal cancer cell line. Asian Pac. J. Cancer Prev. APJCP.

[17] Lee K.E., Shin J.A., Hong I.S., Cho N.P., Cho S.D. (2013). Effect of methanol extracts of *Cnidium officinale* Makino and Capsella bursa-pastoris on the apoptosis of HSC-2 human oral cancer cells. Exp. Ther. Med..

[18] Joo H., Lee H.J., Shin E.A., Kim H., Seo K.H., Baek N.I., Kim B., Kim S.H. (2016). c-Jun N-terminal Kinase-Dependent Endoplasmic Reticulum Stress Pathway is Critically Involved in Arjunic Acid Induced Apoptosis in Non-Small Cell Lung Cancer Cells. Phytother. Res. PTR.

[19] Kim H., Shin E.A., Kim C.G., Lee D.Y., Kim B., Baek N.I., Kim S.H. (2016). Obovatol Induces Apoptosis in Non-small Cell Lung Cancer Cells via C/EBP Homologous Protein Activation. Phytother. Res. PTR.

[20] Kim J., Yun M., Kim E.O., Jung D.B., Won G., Kim B., Jung J.H., Kim S.H. (2016). Decursin enhances TRAIL-induced apoptosis through oxidative stress mediated- endoplasmic reticulum stress signalling in non-small cell lung cancers. Br. J. Pharmacol..

[21] Kyle R.A., Jacobus S., Friedenberg W.R., Slabber C.F., Rajkumar S.V., Greipp P.R. (2009). The treatment of multiple myeloma using vincristine, carmustine, melphalan, cyclophosphamide, and prednisone (VBMCP) alternating with high-dose cyclophosphamide and α_2_β interferon versus VBMCP: Results of a phase III Eastern Cooperative Oncology Group Study E5A93. Cancer.

[22] Facon T., Mary J.Y., Hulin C., Benboubker L., Attal M., Pegourie B., Renaud M., Harousseau J.L., Guillerm G., Chaleteix C. (2007). Melphalan and prednisone plus thalidomide versus melphalan and prednisone alone or reduced-intensity autologous stem cell transplantation in elderly patients with multiple myeloma (IFM 99-06): A randomised trial. Lancet.

[23] Aghajanian C., Soignet S., Dizon D.S., Pien C.S., Adams J., Elliott P.J., Sabbatini P., Miller V., Hensley M.L., Pezzulli S. (2002). A phase I trial of the novel proteasome inhibitor PS341 in advanced solid tumor malignancies. Clin. Cancer Res..

[24] Ailawadhi S., Frank R.D., Sharma M., Menghani R., Temkit M., Paulus S., Khera N., Hashmi S., Advani P., Swaika A. (2018). Trends in multiple myeloma presentation, management, cost of care, and outcomes in the Medicare population: A comprehensive look at racial disparities. Cancer.

[25] Nomura S., Ito T., Yoshimura H., Hotta M., Nakanishi T., Fujita S., Nakaya A., Satake A., Ishii K. (2018). Evaluation of thrombosis-related biomarkers before and after therapy in patients with multiple myeloma. J. Blood Med..

[26] Lee Y.M., Lee Y.R., Kim C.S., Jo K., Sohn E., Kim J.S., Kim J. (2016). *Cnidium officinale* extract and butylidenephthalide inhibits retinal neovascularization in vitro and in vivo. BMC Complement. Altern. Med..

[27] Bae K.E., Choi Y.W., Kim S.T., Kim Y.K. (2011). Components of rhizome extract of *Cnidium officinale* Makino and their in vitro biological effects. Molecules.

[28] Kwak D.H., Kim J.K., Kim J.Y., Jeong H.Y., Keum K.S., Han S.H., Rho Y.I., Woo W.H., Jung K.Y., Choi B.K. (2002). Anti-angiogenic activities of *Cnidium officinale* Makino and Tabanus bovinus. J. Ethnopharmacol..

[29] Cao S.S., Kaufman R.J. (2014). Endoplasmic Reticulum Stress and Oxidative Stress in Cell Fate Decision and Human Disease. Antioxid. Redox Signal..

[30] Belmont P.J., Chen W.J., Thuerauf D.J., Glembotski C.C. (2012). Regulation of microRNA expression in the heart by the ATF6 branch of the ER stress response. J. Mol. Cell. Cardiol..

[31] Yang F., Zhang L., Wang F., Wang Y., Huo X.-S., Yin Y.-X., Wang Y.-Q., Zhang L., Sun S.-H. (2011). Modulation of the unfolded protein response is the core of microRNA-122-involved sensitivity to chemotherapy in hepatocellular carcinoma. Neoplasia.

[32] Dai R., Li J., Liu Y., Yan D., Chen S., Duan C., Liu X., He T., Li H. (2010). miR-221/222 suppression protects against endoplasmic reticulum stress-induced apoptosis via p27Kip1-and MEK/ERK-mediated cell cycle regulation. Biol. Chem..

