# Supplementary material for: miR-211 Plays a Critical Role in Cnidium officinale Makino Extract-Induced, ROS/ER Stress-Mediated Apoptosis in U937 and U266 Cells
Source: Int J Mol Sci. 2018 Mar 15;19(3):865. doi: 10.3390/ijms19030865 (PMC5877726; doi:10.3390/ijms19030865)
Supplement: Supplementary file 1 [file ijms-19-00865-s001.pdf]

miR-211 Plays a Critical Role in *Cnidium officinale* Makino Extract-Induced, ROS/ER Stress-Mediated Apoptosis in U937 and U266 Cells

Jin Ah Cha [1,†], Hyo-Sook Song [2,†], Beomku Kang [1], Moon Nyeo Park [1], Kyoung Sun Park [3], Sung-Hoon Kim [1], Bum-Sang Shim [1,*] and Bonglee Kim [1,2,*]

1 Department of Pathology, College of Korean Medicine, Graduate School, Kyung Hee University, 1 Hoegi-dong, Dongdaemun-gu, Seoul 130-701, Korea; nicesh89@khu.ac.kr (J.A.C.); beomkukang@khu.ac.kr (B.K.); mnpark@khu.ac.kr (M.N.P.); sungkim7@khu.ac.kr (S.-H.K.)

2 Department of Science in Korean Medicine, College of Korean Medicine, Graduate School, Kyung Hee University, 1 Hoegi-dong, Dongdaemun-gu, Seoul 130-701, Korea; shs331@khu.ac.kr

3 Department of Korean Medicine Obstetrics & Gynecology, College of Korean Medicine, Kyung Hee University, 1 Hoegi-dong, Dongdaemun-gu, Seoul 130-701, Korea; lovepks@khnmc.or.kr (K.S.P)

* Correspondence: bongleekim@khu.ac.kr (B.K.); Tel.: +82-2-961-9217 (B.K.)

* Co-correspondence: eshimbs@khu.ac.kr (B.-S.S.); Tel.: +82-2-961-0047 (B.-S.S.)

† These authors contributed equally to this work.

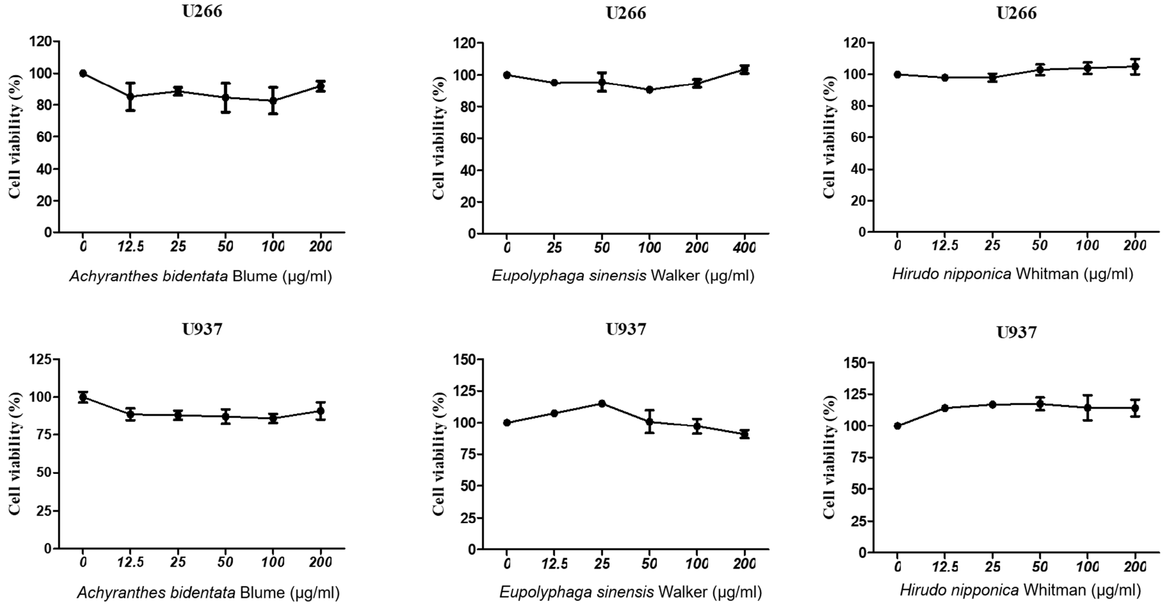

Supplementary Figure S1. Cytotoxic effect of *Achyranthes bidentata* Blume (ABB), *Eupolyphaga sinesis* Walker (ESW) and *Hirudo nipponica* Whitman (HNW) in U937 and U266 cells. Cells were seeded into 96 well microplates at a density of 2 x 10^4 cells/well and treated with various concentrations of ABB, ESW and HNW (0, 12.5, 25, 50, 100 or 200 μg/ml) for 24 h. Cell viability was measured by EZ-cytox Enhanced cell viability assay kit. Values represent the means of 3 experiments ± SD.

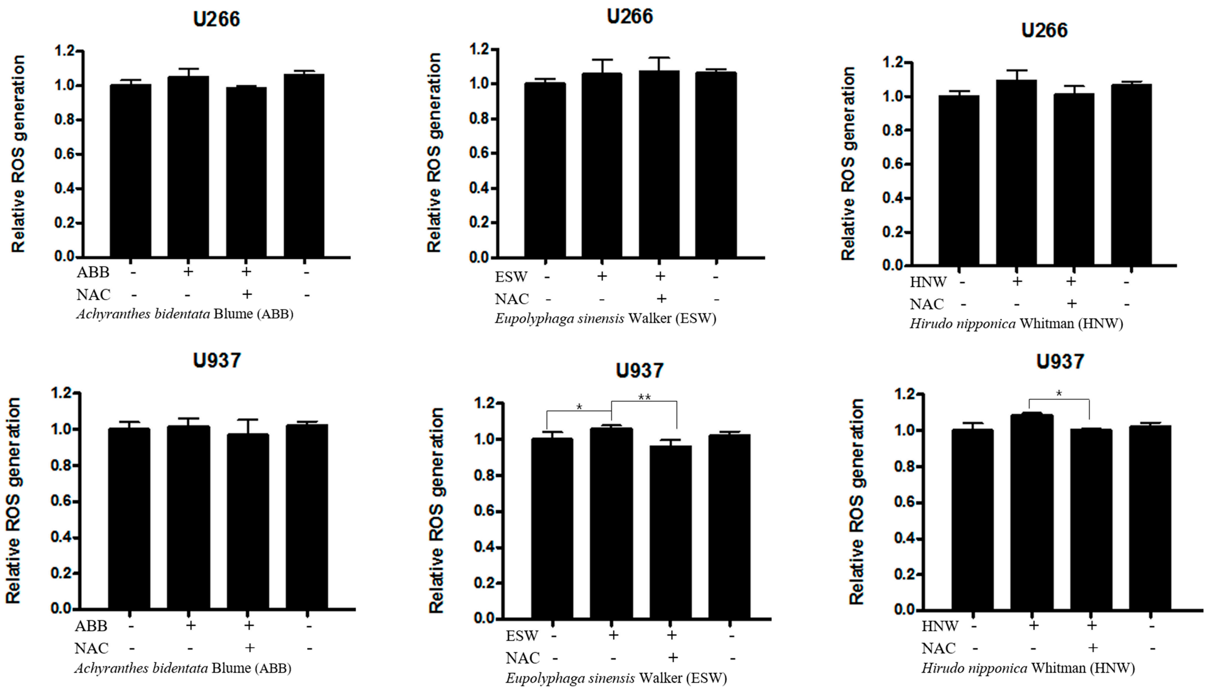

Supplementary Figure S2. The effect of COM on ROS production in *Achyranthes bidentata* Blume (ABB), *Eupolyphaga sinesis* Walker (ESW), *Hirudo nipponica* Whitman (HNW) treated U937 and U266 cells. Cells were treated with indicated extracts (80 μg/ml) for 24 h with or without pre-treatment of NAC (5 mM) for 1 h. ROS production was determined by cellular reactive oxygen species detection assay kit. Values represent the means of 3 experiments ± SD.
